# Specificities of Meningitis and Meningo-Encephalitis After Kidney Transplantation: A French Retrospective Cohort Study

**DOI:** 10.3389/ti.2023.10765

**Published:** 2023-01-18

**Authors:** Y. Tamzali, A. Scemla, T. Bonduelle, C. Garandeau, M. Gilbert, S. Randhawa, T. De Nattes, H. Hachad, V. Pourcher, P. Taupin, H. Kaminski, M. Hazzan, V. Moal, M. Matignon, V. Fihman, C. Levi, M. Le Quintrec, J. M. Chemouny, E. Rondeau, D. Bertrand, E. Thervet, S. Tezenas Du Montcel, E. Savoye, B. Barrou, N. Kamar, J. Tourret

**Affiliations:** ^1^ Sorbonne Université, Assistance Publique-Hôpitaux de Paris (AP-HP), Pitié-Salpêtrière Hospital, Medical and Surgical Department of Kidney Transplantation, Paris, France; ^2^ Sorbonne Université, Assistance Publique-Hôpitaux de Paris (AP-HP), Pitié-Salpêtrière Hospital, Department of Infectious and Tropical Diseases, Paris, France; ^3^ Université Paris-Descartes, Assistance Publique-Hôpitaux de Paris (AP-HP), Department of Nephrology and Kidney Transplantation, Hôpital Necker, Paris, France; ^4^ Neurology Department, Epilepsy Unit, Centre Hospitalier Universitaire de Bordeaux, Bordeaux, France; ^5^ Nephrology Department, Centre Hospitalier Universitaire de Nantes, Nantes, France; ^6^ Nephrology and Transplantation Department, Centre Hospitalier Universitaire de Lille, Lille, France; ^7^ Aix-Marseille Université, Hôpitaux Universitaires de Marseille, Hôpital Conception, Center of Nephrology and Kidney Transplantation, Marseille, France; ^8^ Department of Nephrology Dialysis and Kidney Transplantation, Centre Hospitalier Universitaire de Rouen, Rouen, France; ^9^ University Paris-Descartes, Assistance Publique-Hôpitaux de Paris (AP-HP), Department of Biostatistics, Necker Hospital, Paris, France; ^10^ Department of Nephrology, Transplantation, Dialysis and Apheresis, CHU Bordeaux, Bordeaux, France; ^11^ Université Paris Est Créteil, Institut National de la Santé et de la Recherche Médicale (INSERM), Institut Mondor de Recherche Biomédicale (IMRB), Créteil, France; ^12^ Assistance Publique des Hôpitaux de Paris (AP-HP), Hôpitaux Universitaires Henri Mondor, Service de Néphrologie et Transplantation, Fédération Hospitalo-Universitaire, Innovative Therapy for Immune Disorders, Créteil, France; ^13^ Bacteriology and Infection Control Unit, Department of Prevention, Diagnosis, and Treatment of Infections, Henri-Mondor University Hospital, Assistance Publique des Hôpitaux de Paris (AP-HP), Créteil, France; ^14^ EA 7380 Dynamyc, EnvA, Paris-Est University (UPEC), Créteil, France; ^15^ Department of Nephrology Immunology and Kidney Transplantation, Centre Hospitalier Univeristaire Edouard Herriot, Lyon, France; ^16^ Department of Nephrology Dialysis and Kidney Transplantation, Centre Hospitalier Universitaire de Montpellier, Montpellier, France; ^17^ Université de Rennes, CHU Rennes, INSERM, EHESP, IRSET—UMR_S 1085, CIC‐P 1414, Rennes, France; ^18^ Sorbonne Université, Assistance Publique-Hôpitaux de Paris (AP-HP), Department of Nephrology, SINRA, Hôpital Tenon, GHEP, Paris, France; ^19^ Université Paris-Descartes, Assistance Publique-Hôpitaux de Paris (AP-HP), Department of Nephrology, Hôpital Europeen Georges Pompidou, Paris, France; ^20^ Sorbonne Université, INSERM, Pierre Louis Epidemiology and Public Health Institute, Assistance Publique-Hopitaux de Paris (AP-HP), Medical Information Department, Pitié Salpêtrière-Charles Foix University Hospital, Paris, France; ^21^ Agence de la Biomédecine, Saint Denis, France; ^22^ Sorbonne Université, Assistance Publique-Hôpitaux de Paris (AP-HP), Pitié-Salpêtrière Hospital, Medical and Surgical Department of Kidney Transplantation, INSERM, UMR 1082, Paris, France; ^23^ Department of Nephrology and Organ, INFINITY-INSERM U1291-CNRS U5051, Université Paul Sabatier, Toulouse, France; ^24^ Sorbonne Université, Assistance Publique-Hôpitaux de Paris (AP-HP), Pitié-Salpêtrière Hospital, Medical and Surgical Department of Kidney Transplantation, INSERM, UMR 1138, Paris, France

**Keywords:** kidney transplantation, immunosuppression, transplant infectious diseases, opportunistic infections, meningitis, encephalitis, *Cryptococcus neoformans*, enterobacteriales

## Abstract

Kidney transplant recipients develop atypical infections in their epidemiology, presentation and outcome. Among these, meningitis and meningoencephalitis require urgent and adapted anti-infectious therapy, but published data is scarce in KTRs. The aim of this study was to describe their epidemiology, presentation and outcome, in order to improve their diagnostic and management. We performed a retrospective, multicentric cohort study in 15 French hospitals that included all 199 cases of M/ME in KTRs between 2007 and 2018 (0.9 case per 1,000 KTRs annually). Epidemiology was different from that in the general population: 20% were due to *Cryptococcus neoformans*, 13.5% to varicella-zoster virus, 5.5% to *Mycobacterium tuberculosis*, and 4.5% to Enterobacteria (half of which produced extended spectrum beta-lactamases), and 5% were Post Transplant Lymphoproliferative Disorders. Microorganisms causing M/ME in the general population were infrequent (2%, for *Streptococcus pneumoniae*) or absent (*Neisseria meningitidis*). M/ME caused by Enterobacteria, *Staphylococci* or filamentous fungi were associated with high and early mortality (50%–70% at 1 year). Graft survival was not associated with the etiology of M/ME, nor was impacted by immunosuppression reduction. Based on these results, we suggest international studies to adapt guidelines in order to improve the diagnosis and the probabilistic treatment of M/ME in SOTRs.

## Graphical Abstract

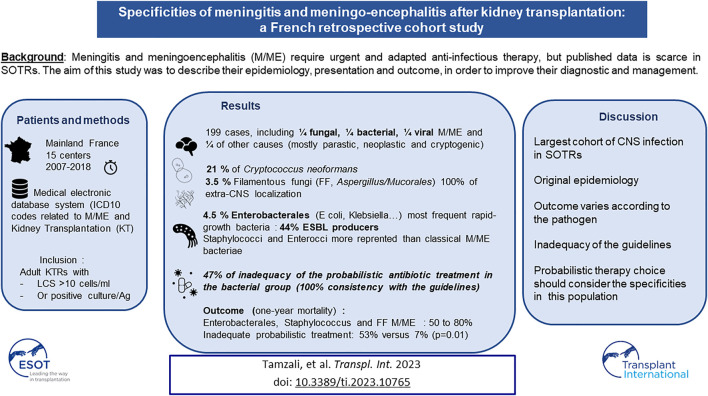

## Introduction

Meningitis and meningo-encephalitis (M/ME) are potentially life-threatening infections with causes that are well described in immunocompetent hosts (1-3). According to current guidelines (4-7), clinical suspicion of M/ME implies the rapid initiation of high-dose, broad-spectrum probabilistic anti-infectious therapies while performing radiological and cerebrospinal fluid (CSF) biological work-up (8). Solid Organ Transplant Recipients (SOTRs) are subject to invasive infectious diseases, sometimes with atypical and severe presentation, and with a wider range of pathogens than the general population (5-11). A knowledge of the specific epidemiology of central nervous system (CNS) infections in this population is therefore critical to elicit the best probabilistic anti-infectious therapy. Non-etheless, few studies describe the epidemiology, clinical presentation and outcome of M/ME in SOTRs: retrospective cohorts of specific pathogens (12-15), mixed cohorts of liver, heart, and kidney transplant (KT) recipients (KTRs) (10-21) and case reports (22-27).

Here we describe the epidemiology, presentation, and outcome of M/ME that occurred in KTRs in France between 2007 and 2018, with a 2-year follow-up.

## Patients and Methods

### Study Population

KTRs diagnosed with M/ME between 1st January 2007, and 31st December 2018, were identified in fifteen French Academic Hospitals with a kidney transplantation program. Participating centers were the university hospitals from the following French cities: Bordeaux, Lille, Limoges, Lyon, Marseille Montpellier, Nantes, Paris (Georges Pompidou, Henri Mondor, Necker, Pitié-Salpêtrière, Tenon), Rennes, Rouen and Toulouse.

The national electronic medical databases of these centers were screened for the International Statistical Classification of Diseases and Related Health Problems 10th Revision (ICD-10) codes for kidney transplantation and for codes related to CNS infections and their main etiologies (see Supplementary Material). When available, local clinical and microbiology databases were also screened.

The inclusion criteria were:

- Adult (≥18 years old) KTRs
- Diagnosis of M/ME between 1st January 2007 and 31st December 2018 defined by at least one of these observations:
  * CSF pleocytosis over 10 cells/mm^3^
  * Positive CSF bacterial or mycological culture
  * Positive CSF antigen (*C. neoformans* [CrAg], *Aspergillus*, or *Streptococcus pneumoniae*).
- A functioning kidney graft at the diagnosis of M/ME

Consistent with these inclusion criteria, non-infectious meningitis were included.

Exclusion criteria:

- CSF with a positive polymerase chain reaction (PCR) for a pathogen without hypercellularity
- Cerebral abscess without CSF hypercellularity
- Subarachnoid hemorrhage
- High CSF protein concentration without hypercellularity

The following data were collected from the medical charts:

- Medical history, characteristics of the KT, immunosuppressive therapy and anti-infectious prophylaxis protocols.
- Clinical, biological, microbiological and radiological presentation at admission for M/ME.
- Therapeutic management, including anti-infectious and surgical treatments but also immunosuppressive treatment modulation, *i.e.*, a change of therapeutic class, the discontinuation of a drug, or a decrease of at least 25% of the trough level target.
- Clinical and biological outcome after meningitis, including patient and graft survival. The data was collected until the last available follow-up.

Meningitis was defined as the presence of cerebrospinal pleocytosis >10 elements/mm^3^.

Encephalitic features were defined as the presence of one of the following: mental status or cognitive impairment, generalized seizures. Meningo-encephalitis was defined as the association of meningitis and encephalitic features.

A CSF was defined as lymphocytic or neutrophilic if its cellularity was made up of >50% of lymphocytes or neutrophils, respectively. It was defined as mixed if the difference between these percentages was below 10%.

Causative diagnosis was asserted by an infectious disease specialist based on specific chart review.

The “highly immunosuppressed status” was defined as the presence of at least one the following criteria:

- Recent KT (<6 months)
- A history of immunosuppressive therapy before KT
- A history of treatment of a rejection episode between last KT and the M/ME onset
- A recent (<2 years before M/ME) cytotoxic chemotherapy treatment
- Recent history of hemopathy (<5 years)

### Statistical Analysis

The statistical analysis was performed using GraphPad PRISM^®^ v9 (GraphPad Software, San Diego, CA, United States).

The annual incidence of M/ME was estimated by dividing the number of yearly cases by the number of living KTRs in the 15 participating centers during the same year.

Quantitative variables are presented as mean ± standard deviation or median (inter-quartile range, IQR) according to their normal or skewed distributions. Qualitative variables are presented as numbers (percentages). Data were compared using the Student’s t-test, the Mann-Whitney test, or the χ^2^ test as appropriate. In analysis including more than two groups, the data were compared using one-way ANOVA or Kruskal-Wallis test according to their normal or skewed distributions.

Survival analyses were performed using the Kaplan-Meier method. A Log-rank test was performed for the comparison between the groups, with a significant *p*-value of < 0.05.

### Ethics

This study was approved by the Paris Public Hospitals (“Assistance Publique-Hôpitaux de Paris”) Office of Data Protection (RGPD) and registered under project number 20181105112928.

## Results

### Incidence of M/ME

**FIGURE 1:**
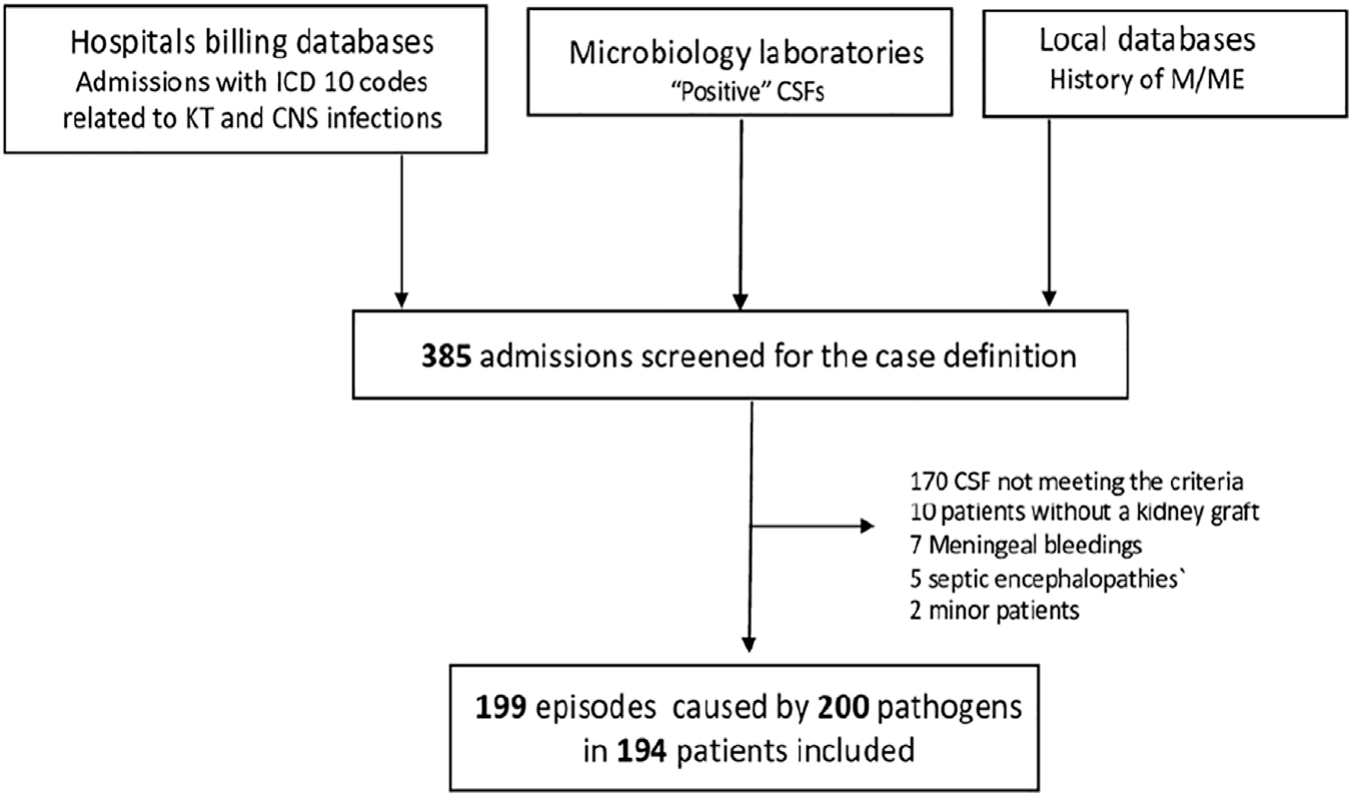
Flow Chart. ICD, international classification of diseases; CNS, central nervous system; CSF, cerebrospinal fluid; M/ME, meningitis/meningo-encephalitis.

Between 2007 and 2018, 199 cases of M/ME, caused by 200 pathogens were diagnosed in 194 patients (Figure 1), with a median follow-up of 3.58 IQR [10.0–69.0]. The mean annual incidence was 0.9 for 1,000 KTRs with no significant variation over the study period (*p* = 0.81).

### Population Characteristics

The characteristics of the patient population are shown in Table 1. M/ME mostly involved male patients (60.3%), born in mainland France (70.4%), at a mean age of 54.8 ± 14.4 years, and transplanted for the first time (82% of the cases). Thirty-six percent of the patients were also treated for diabetes mellitus, which was the most frequent cause of the initial nephropathy. Induction therapy consisted of anti-thymocyte therapy in two-thirds of the patients (consistently with previous French study reporting 41%–75% of anti-thymocyte therapy induction according to centers) (28) and almost half of the cohort (47%) matched our definition of highly immunosuppressed (see *Patients and Methods*). No patients had been treated with eculizumab or any other anti-complement therapy.

**TABLE 1:** Characteristics of the population.

Characteristic	*N* = 194
	n (%) or mean ± SD
Age (yr)	54.8 ± 14.4
Gender, (males)	117 (60.3)
Dialysis duration before KT, (yr)	4.9 ± 4.6
Previous KT	
None	159 (82.0)
1	31 (16.0)
≥2	4 (2.0)
Donor	
Deceased	173 (89.2)
Living	21 (10.8)
Country of birth	
Mainland France	136 (70.4)
North Africa	21 (10.6)
Subsaharian Africa	18 (9.0)
Other	18 (9.0)
Initial nephropathy	
Diabetes	29 (14.9)
CTIN	22 (11.3)
Undetermined	22 (11.3)
Polycystic Kidney Disease	18 (9.2)
Hypertensive nephropathy	18 (9.2)
IgA AN	17 (8.7)
Other	69 (35.6)
Diabetes	71 (36.6)
Pre-existing	41 (21.1)
Post-transplantation	30 (15.5)
Anti-infectious prophylaxis at M/ME onset	
Cotrimoxazole	41 (20.6)
Valaciclovir	32 (16.1)
Valganciclovir	7 (3.5)
Antifungal (azoles)	0 (0.0)
Immunity status	
Anti-thymocyte globulins for induction	113 (58.5)
HIV seropositivity	6 (3.0)
Highly immunosuppressed before M/ME^a^	91 (47.0)
History of treated rejection	51 (25.9)
Other	146 (74.1)
eGFR^b^ at M/ME (ml/min/1.73 m^2^)	46.5 ± 26.8

^a^ Defined as the presence of at least on the following: recent KT (<6 months), history of immunosuppressive therapy before KT, history of treatment of a rejection episode before M/ME onset, recent (<2 years) cytotoxic chemotherapy treatment, hemopathy.

^b^ Most recent estimated glomerular filtration rate considered as stable before the onset of the M/ME, calculated according to the Modification of Diet in Renal Disease study equation.

CTIN, Chronic Tubulo-interstitial Nephropathy; IgA AN, IgA associated nephropathy.

### Etiologies of the M/ME in KTRs

**FIGURE 2:**
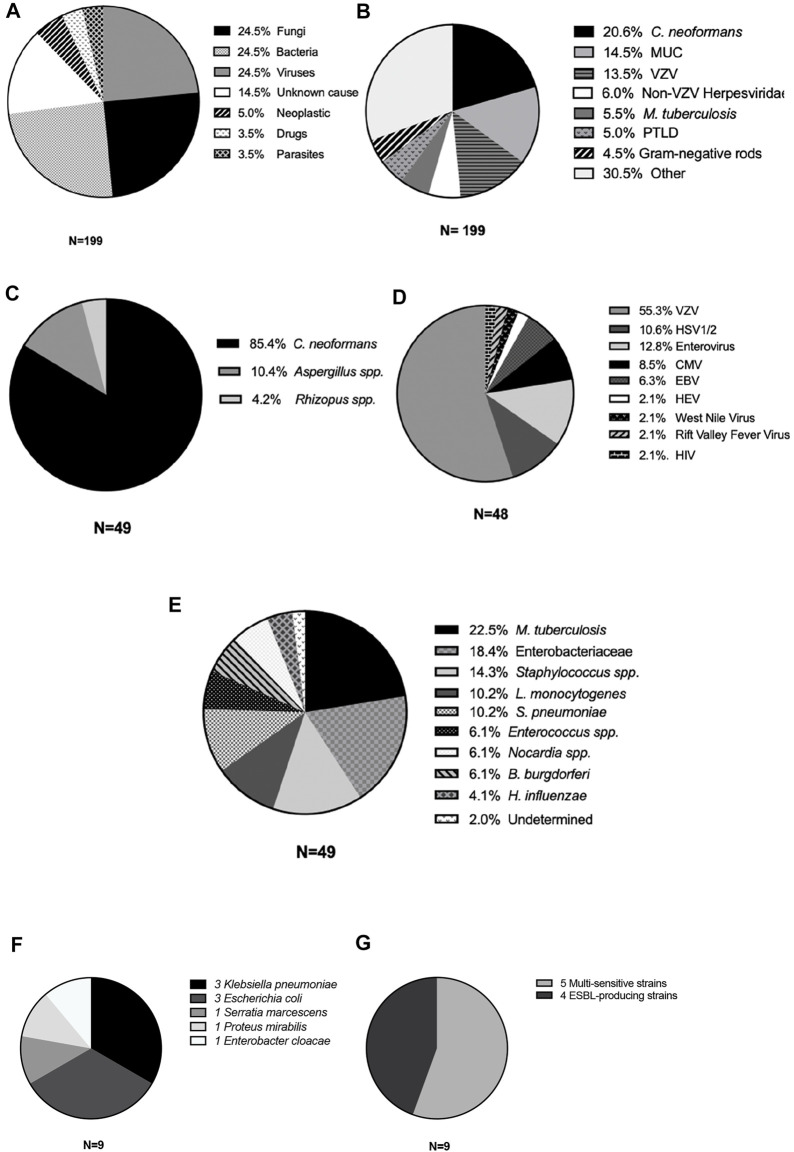
Causes of meningitis and meningoencephalitis in kidney transplant recipients. Cases of M/ME are represented according to type of etiology **(A)**, main microorganism or cause **(B)**, type of Fungi **(C)**, virus **(D)**, or bacteria **(E)**. **(F)** All Enterobacteriales; **(G)** proportion of extended spectrum beta-lactamase producing Enterobacteriales. VZV, varicella-zoster virus; MUC, meningitis of unknown cause; VZV, varicella-zoster virus; PTLD, post-transplant lymphoproliferative disorder; HSV, herpes simplex virus; CMV, cytomegalovirus; EBV, Epstein-Barr virus; HEV, hepatitis E virus; HIV, human immunodeficiency virus; ESBL, extended-spectrum betalactamase.

Causes of M/ME were almost homogeneously distributed between Fungi, Bacteria, and Viruses (one-quarter each). The last quarter was divided into parasitic, non-infectious M/ME and M/ME of unknown cause (MUC) (Figure 2A).

Overall, the most frequent microorganisms were *C. neoformans* (20%), VZV, 13.5%, *Mycobacterium tuberculosis* (5.5%), Enterobacterales (4.5%) and filamentous Fungi (4.0%), Figure 2B and Supplementary Table S2. In 29 patients (totaling 14.5% of M/ME episodes) the cause remained unknown even after extensive investigation, making MUC the second-most frequent diagnosis. We compared the distribution of the main etiologies according to the level of immunosuppression (Supplementary Table S4). There was no striking difference except from filamentous fungi infections, all occurring in the highly immunosuppressed group (8 versus 0, *p* = 0.002).

### Delay Between KT and M/ME

Half of the cases occurred within the first 3.4 (IQR [0.91–8.58]) years after KT. The earliest episode was diagnosed on the first day after KT and the latest case was diagnosed 43 years after transplantation.

The delay before M/ME onset varied with the group of etiology: viral M/ME occurred after a median delay of 2.5 years (QR 0.7–8.8), fungal M/ME after 2.8 years (IQR 0.7–5.8), bacterial M/ME after 3.5 years (IQR 1.3–8.8), parasitic M/ME after 4.9 years (IQR 2.0–9.8) and M/ME due to non-infectious causes after a median delay of 9.1 years (2.0–11.8).

**FIGURE 3:**
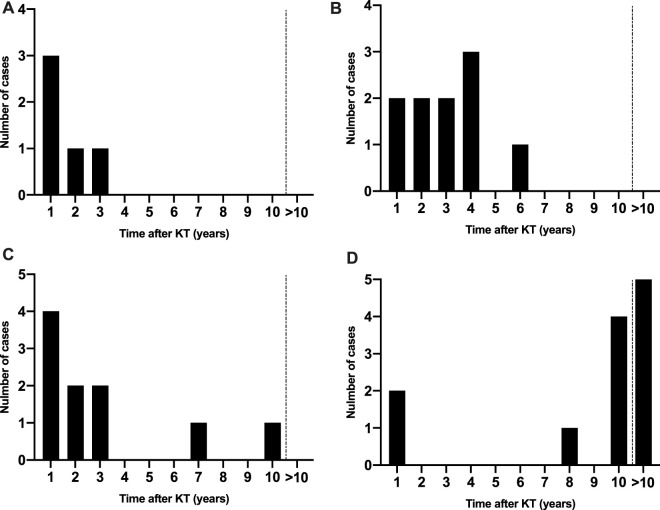
Distribution of M/ME over time after KT according to the etiology. **(A)** Cytomegalovirus cases, **(B)** Gram-negative rod cases, **(C)** Filamentous fungi, **(D)** Post-transplant lymphoproliferative disorders.

The incidence of M/ME after KT was not linear for all microorganisms and varied according to the cause of MME. M/ME due to CMV and filamentous fungi occurred in the first 3 years after KT (Figures 3A, C), and Gram-negative rods (GNR) M/ME occurred in the first 4 years after KT in 90% of the cases (Figure 3B). PTLD (Post-Transplant Lymphoproliferative Disease) was the first cause of M/ME after 10 years post-transplantation (Figure 3D). The other etiologies, and especially *C. neoformans* and VZV did not seem to vary in risk in the years following KT.

### Clinical and Biological Presentation at Diagnosis

The clinical presentation at admission is described in Table 2. The patients with a bacterial or a fungal M/ME presented more frequently with fever (85.4% and 87.5% versus 26%–72.1% in the other groups, *p* = 0.0006) and the patients with fungal M/ME more frequently with headaches (95.8% versus 42.8%–82.4%, *p* = 0.001). Neck stiffness was observed more frequently in bacterial and fungal infections (55% and 52%) than in viral infections (23%, *p* = 0.08).

**TABLE 2:** Clinical, biological and radiological characteristics of M/ME in the different etiological groups.

	All (194)	Fungi (49)	Viruses (48)	Bacteria (49)	Parasites (7)	Non-infectious (17)	*p*
	n/N (%) or median [IQR]	n/N (%) or median [IQR]	n/N (%) or median [IQR]	n/N (%) or median [IQR]	n/N (%] or median [IQR]	n/N [%] or median [IQR]	
Fever	146/193 (75.6)	41/48 (85.4)	31/43 (72.1)	43/49 (87.8)	2/7 (26.6)	9/17 (52.9)	0.0006
Headaches	151/192 (78.7)	46/48 (95.8)	32/43 (74.4)	39/49 (79.6)	3/7 (42.8)	14/17 (82.4)	0.009
Neck stiffness	73/193 (37.8)	23/48 (47.9)	10/43 (23.3)	11/49 (22.5)	1/7 (14.3)	6/17 (42.9)	0.03
Clinical encephalitis	94/193 (47.5)	26/48 (54)	26/43 (60.4)	35/49 (71.4)	3/7 (42.8)	4/17 (23)	0.01
Seizures	31/193 (16.0)	6/48 (12.5)	4/43 (9.3)	11/49 (22.4)	0/7 (0)	2/17 (11.8)	<0.0001
Abnormal EEG	12/90 (42.9)	6/16 (37.5)	16/23 (69.6)	14/20 (70)	3/4 (75)	4/6 (66.6)	0.23
Extra-CNS involvement	98/194 (53.3)	27/49 (55.1)	26/43 (60.5)	30/49 (61.2)	2 (26.6)	7/17 (41.2)	0.16
Last total lymphocyte count, elts/mm^3^	880 [500–1,387]	690 [430–1,100]	1,199 [700–1,400]	880 [500–1,450]	750 [380–880]	1,360 [404–1,520]	0.08
Last CD4^+^ count, elts/mm^3^	234 [95–529]	158 [90–317]	278 [147–539]	225 [77–459]	448 [251–762]	527 [228–782]	0.02
Leucocyte count at admission, ×1,000 elts/mm^3^	7.0 [3.9–10.0]	6.3 [3.6–9.7]	7.0 [4.6–102.5]	8.6 [6.0–14.8]	7.0 [4.0–7.6]	5.0 [3.0–7.1]	0.01
CRP at admission, mg/L	15.6 [5.0–82.0]	21 [5.0–80.5]	6.5 [2.5–27.8]	113 [56–209]	3.5 [3.0–7.0]	7 [5.0–17.5]	<0.0001
CSF cell count, elts/mm^3^	53 [16–220]	45 [12–171]	6.5 [12–129]	113 [32–759]	11 [10–20]	84 [30–305]	0.0006
Lymphocytic	113 (60.1)	20 (45.5)	38 (90.5)	19 (39.6)	6 (85.7)	10 (58.8)	
Neutrophilic	57 (30.6)	19 (43.2)	3 (7.1)	26 (54.2)	0 0)	5 (29.4)	<0.001
Mixed	16 (8.1)	5 (11.3)	1 (2.3)	3 (6.1)	1 (14.3)	2 (11.8)	
CSF proteins, g/L	0.9 [0.6–1.8]	0.1 [10.6–2.0]	1.6 [0.5–1.4]	2.3 [0.7–3.5]	0.7 [0.4–1.1]	0.8 [0.5–1.0]	0.04
CSF blood/glucose ratio	0.5 [0.4–0.6]	0.5 [0.3–0.6]	0.6 [0.5–0.6]	0.4 [0.3–0.5]	0,7 [0.4–1.1]	0.5 [0.5–0.7]	0.0003
Abnormal findings on CT-scan	38/153 (25.2)	13/43 (30.2)	1/27 (3.7)	12/37 (32.4)	6/7 (85.7)	3/13 (23.1)	0.0005
Abnormal findings on MRI	93/151 (62.8)	26/40 (65.0)	20/34 (58.8)	22/32 (68.8)	7/7 (100)	8/13 (61.5)	0.8

EEG, electroencephalography; CRP, C-reactive protein; CSF, cerebro-spinal fluid; CT-Scan, computed tomography scanner; MRI, magnetic resonance imaging.

Almost half of the patients presented clinical and/or encephalographic encephalitis. In more than half of the cases (53.3%), the clinical presentation included extra-CNS manifestations that could facilitate the diagnosis. The extra-neurologic involvements associated with the most frequent causes are summarized in Supplementary Table S3.

**FIGURE 4:**
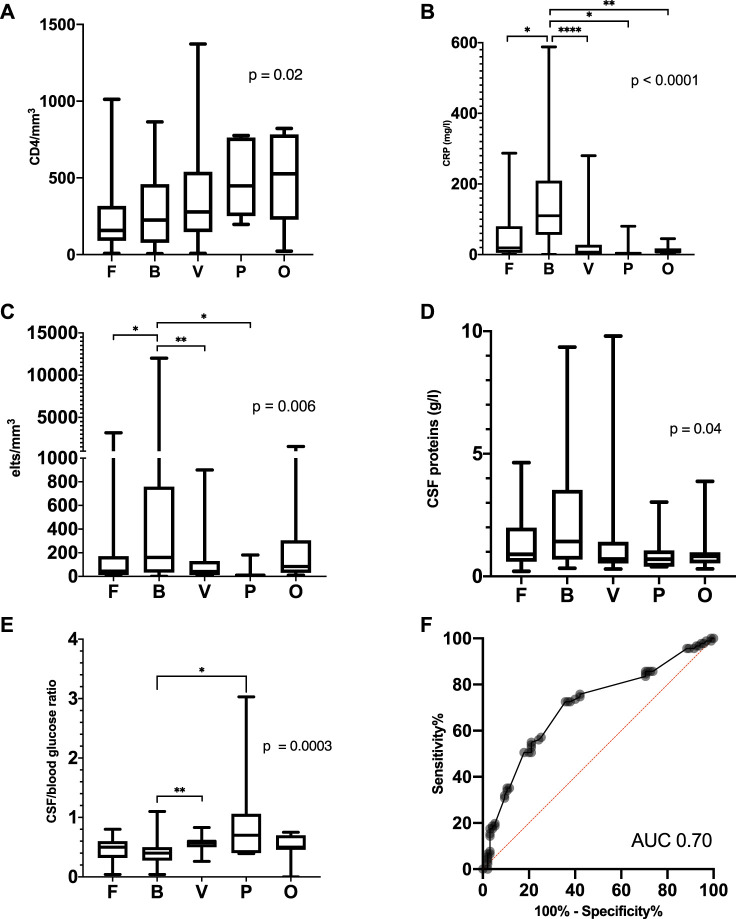
Biological characteristics in the different groups of microorganisms or causes. **(A)** Last CD4^+^ count before M/ME onset, **(B)** C-reactive protein at admission for M/ME, **(C)** CSF cellularity, **(D)** CSF protein level, **(E)** glucose CSF/blood ratio and **(F)** ROC Curve of the glucose CSF/blood ratio to discriminate bacterial and fungal M/ME from the other M/ME. Elts, elements; F, fungi; B, bacteria; V, viruses; P, parasites; O, other.

The number of total lymphocytes did not differ between the etiologic groups, but the CD4^+^ lymphocyte count before onset was lower in the fungal, bacterial and viral group (when taken altogether) than in the parasitic and non-infectious group (Table 2; Figure 4A). Bacterial M/ME resulted in higher CSF cellularity, a higher CSF protein concentration, and a higher serum C-reactive protein concentration than the other M/ME (Figures 4B–F).

The CSF-to-blood glucose ratio was not significantly different between bacterial and fungal M/ME but was lower in bacterial M/ME as compared to Viral and Parasitic forms (*p* = 0.004) and in bacterial and fungal M/ME M/ME compared to the rest of the group (*p* < 0.0001). We assessed the efficiency of various ROC (receiver operating characteristic) curves of the blood to CSF glucose ratio to isolate specific etiologies. The best performance was achieved with a value of 0.5 in discriminating between bacterial and fungal forms vs. the others (area under the curve 0.70, 95% CI [0.63–0.78]) with a sensitivity of 72.5 95% CI [62.6–80.6] and specificity of 64.2 95% CI [54.2–73.1] (Figure 4F).

Viral and parasitic M/ME were lymphocytic in most of the cases, while bacterial M/ME was mostly neutrophilic (Table 2). There were no neutrophilic M/ME in the group of parasitic M/ME. The other groups of M/ME showed no specific cellularity.

### Description of M/ME by Etiology

#### Viruses

Herpesviridae were responsible for 80% of the viral M/ME (Figure 2D), with 55% of VZV infection (all but one were recurrences). Only 4/39 (10%) of the patients with viral M/ME received valganciclovir (VGC) prophylaxis at meningitis onset. The 35 patients without VGC prophylaxis were successfully treated by intravenous acyclovir or ganciclovir, according to the Herpesviridae. Four patients were on VGC prophylaxis when the M/ME declared: one patient developed ganciclovir-resistant CMV infection and died of the meningoencephalitis, and three patients developed HSV or VZV acyclovir-sensitive M/ME, for which they were successfully treated.

Three patients developed EBV (Epstein-Barr Virus) meningo-encephalitis without lymphoproliferative disorder.

#### Bacteria

##### Slow-Growth Bacteria

Eleven patients were diagnosed with tuberculous meningitis, of which only two had a proven diagnostic (negative direct examination, positive culture). Among them, 6/11 (45%) were born outside of Western Europe (mostly in Africa) compared with 58/188 (30%) in the rest of the cohort (*p* = 0.79).

The eleven tuberculous meningitis were lymphocytic (100%), with a CSF-to-blood glucose ratio <0.5 in 72% of the cases and a protein concentration in the CSF >1 g/L in 72% of the cases.

One-year survival after presumptive treatment was 72%.

Three cases of Nocardia infection were included (*N. farcinica*, *N. nova*, and *N. paucivorans* infections). All three of them were brain-space occupying lesions. Two of them were associated with pulmonary lesions that helped the diagnosis. Treatment was medical only, with an association of meropenem and cotrimoxazole or levofloxacin.

##### Pyogenic Bacteria

All seven cases of M/ME occurring within the first year after KT were due to pyogenic bacteria. Listeria (in two patients), ESBL-producing enterobacterales (in two patients), Staphylococci (in two patients) and Entercococcus (one patient) were found as causative pathogens.

The most frequently found rapid-growth bacteria were GNRs (Figure 2E), found in nine patients (20% of bacterial M/ME and 4.5% of all M/ME), including three *Klebsiella pneumoniae*, three *Escherichia coli*, one *Serratia marcescens*, one *Enterobacter cloacae*, and one *Proteus mirabilis* (Figure 2F). Four of the strains (44%) expressed an Extended-Spectrum Beta-lactamase (Figure 2G).

Out of the nine patients with GNRs M/ME, three suffered a urinary tract infection with the same pathogen, and two had positive blood cultures. Three cases out of nine were hospital acquired infections (33%). Six- and 12-month mortality was 33%, and 44% respectively.

Two *Klebsiella* strains isolated from M/ME were retrospectively screened for specific virulence factors (29); none of the seven virulence factors tested were found.

All five cases of *S. pneumoniae* meningitis were related to the contiguous spread of a local infection (two otitis media, one mastoiditis, one ethmoidal sinusitis and one post-surgical breach).

*Staphylococcus* infections were mainly due to *S. aureus* (5/7, including three infective endocarditis). All cases of Staphylococcal M/ME were community acquired. No case of pyogenic M/ME initially presented with septic shock.

The thirty-two patients presenting a M/ME due to a rapid growth bacterium (i.e., excluding *Mycobacteria* and *Nocardia*) were initially all treated with a combination of a third-generation cephalosporin and amoxicillin. Among them, 15 patients (47%) were infected with a bacterium that was not sensitive to this combination. Mortality in the first month was 1/17 (6.7%) for the patients who received an effective treatment and 8/15 (53.3%) for the patients who presented a pathogen that was resistant to the initial therapy, *p* = 0.01.

#### Fungi

The most frequent fungus was *C. neoformans* (41 cases, 20.6% Figures 2B, C), with 95% of the cases presenting with headaches, 83% with fever and 49% with neck stiffness. Upon admission, C-reactive protein ranged from 3 to 287 mg/L, total leucocytes count from 2,300 to 17,800/mm^3^ and CSF analysis showed lymphocytic hypercellularity in 49% of the cases and a cellularity ranging from 0 to 1,350 elements/mm^3^ (median 41 IQR [13–139]).

CSF direct examination (India-Ink stain) had a 51% sensitivity, CSF culture 78% (blood 34%) and CSF Cryptococcal Antigen 94% (blood 93%).

*Aspergillus* and *Mucorales* CNS infections were associated with another localization (sinus and/or lung) in all the patients (Supplementary Table S2).

#### Parasites

Parasitic M/ME were all caused by *Toxoplasma gondii*, presenting as a pauci-cellular (<20 cells/mm^3^) lymphocytic meningitis with a higher CSF-to-blood glucose ratio than in the case of other microorganisms (Figure 4). Cranial CT-scan was abnormal in 71% of the cases, and MRI in 100% of the cases, showing multiple cerebral abscesses.

#### Other Causes

Among the 17 non-infectious causes (Figure 2) 10 were neoplastic and 7 drug-related.

Neoplastic M/ME were characterized by a late onset (11 years after KT IQR [37–141]) mostly due to EBV-related PTLD (8/10).

Intravenous immunoglobulins (5/7), tacrolimus (1/7) and sirolimus were found as possible causes of drug-related meningitis, diagnosed after extensive etiological investigation (exclusion of other possible causes) and after a pharmacovigilance investigation.

### Survival

**FIGURE 5:**
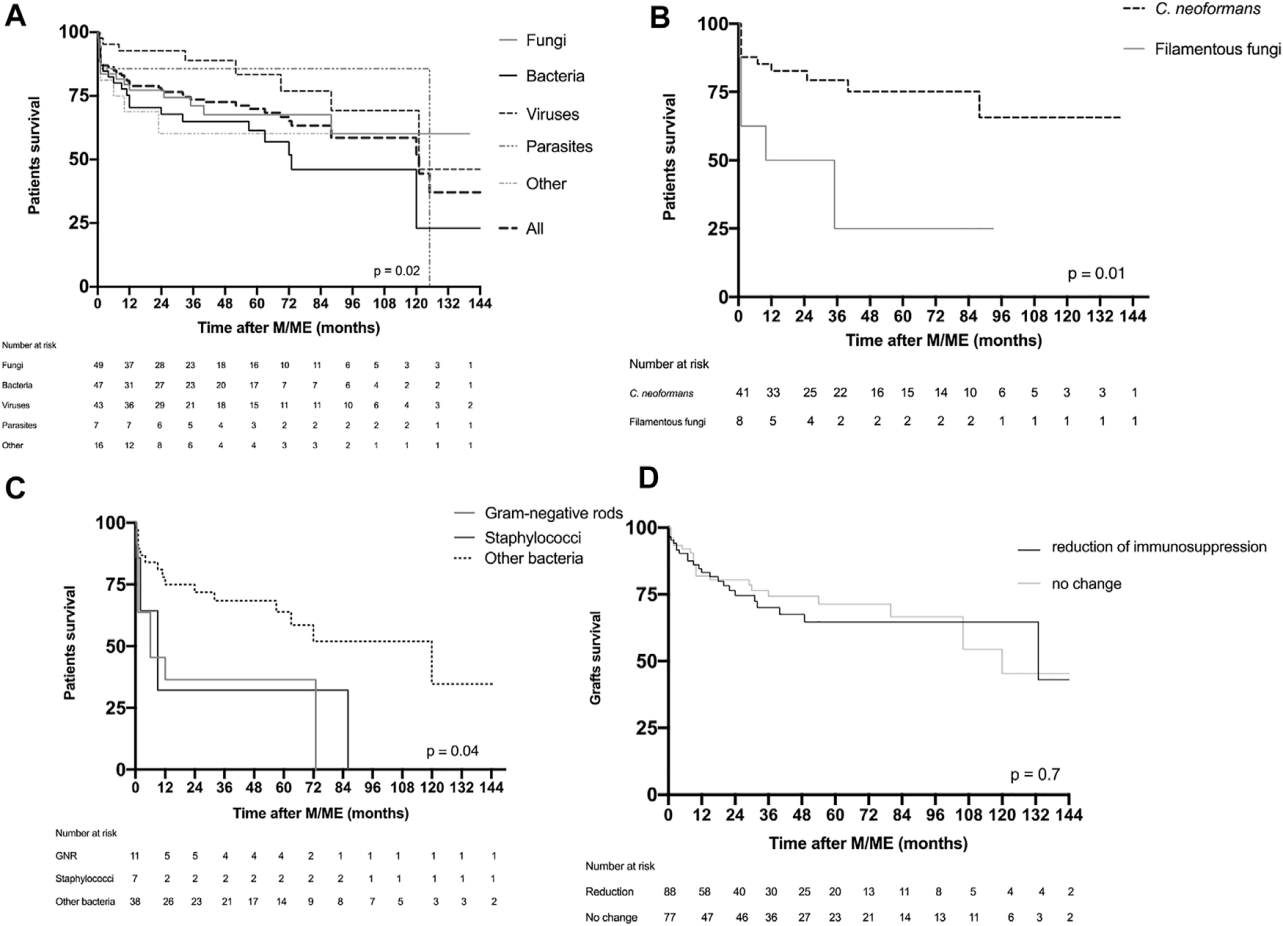
Patient and graft survival. **(A)** Patient survival in the global population presented according to the group of causative microorganism or cause. **(B)** Survival of patients suffering fungal M/ME. **(C)** Survival of patients presenting with bacterial M/ME. **(D)** Graft survival according to reduction of immunosuppression of M/ME. F, fungi; B, bacteria; V, viruses; P, parasites; O, others; CN, *Cryptococcus neoformans*; FF, filamentous fungi; GNR, Gram-negative rods.

One-year and 10-year post-M/ME patient survival were 74% and 70%, respectively (Figure 5A). There were significant differences in the outcome after M/ME according to the etiology Within the fungal group filamentous fungi were associated with the poorest outcome with a mortality of 75% at 36 months (Figure 5B). Within the bacterial group, pyogenic bacterial M/ME 1-year mortality was 57%. GNR and Staphylococcal meningitis were characterized by a particularly high and early mortality (55% and 70% at 6 months, respectively, Figure 5C). *Staphylococcus aureus* M/ME 1 year mortality reached 80%. One-year survival in the group of M/ME of unknown cause was 85%. There was no difference in matter of survival according to the level of immunosuppression.

One-year and 5-year death-censored graft survivals were 82% and 67%, respectively, in the overall population. Mortality was not significantly different between the different etiological groups, nor between the groups of patients undergoing immunosuppression minimization or not (Figure 5D).

## Discussion

We report the largest multicentric study of M/ME in KTRs, including nearly two hundred cases. We show that M/MEs are evenly caused by a wide array of bacteria, fungi and viruses, some of them characterized by specific clinical and/or biological parameters, most of them usually not found in immunocompetent hosts. We also show that the outcome principally depends on the etiology. For these reasons, we believe that the common guidelines for the treatment of M/ME in immunocompetent patients do not apply to KTRs. Consistently with a recent Swiss national study focusing on CNS in SOTs in general (21), M/ME is a relatively rare complication after KT.

Our study population did appeared as highly immunocompromised. We could not search for risk factors as we chose not to perform a controlled study to favor more numerous inclusions according to our goal.

This study reveals several unique features of the M/ME epidemiology in KTRs. First of all, *C. neoformans*, VZV, Enterobacterales, and *Mycobacterium tuberculosis* totalize more than half of the cases. There were fewer than 4% of *S. pneumoniae* and *Haemophilus influenzae*, and no case of *Neisseria meningitidis*. Only five patients (2.5%) were diagnosed with *L. monocytogenes* infection. This strongly contrasts with the epidemiology of M/ME found in the general population (1, 2, 4).

Cotrimoxazole (CMX) prophylaxis could be one of the reasons for this strikingly different epidemiology (9). In KTRs receiving CMX prophylaxis, no *L. monocytogenes*, *Staphylococcus*, *S. pneumoniae* or *Nocardia* M/ME were observed. One case of GNR M/ME occurred while the patient was on CMX prophylaxis, but he was infected with a CMX-resistant ESBL-producing *Klebsiella pneumoniae* strain. One case of *Toxoplasma gondii* infection was also observed despite ongoing CMX prophylaxis.

We could not study the role of anti-pneumococcal vaccination in our population as this was optimized and recommended in France during the study period. In addition, its effectiveness remains controversial in SOTRs (30).

MUCs were frequent in our cohort (29 patients, 14.5% of the cases) consistent with what has been found previously (31). Most of these cases received a final diagnosis of a “possible viral origin” as they were frequently self-limiting. However, 72% of the patients received probabilistic anti-infectious therapy as recommended by the guidelines (consisting of an association of high dose cephalosporin 200 mg/kg/d, amoxicillin 200 mg/kg/d and acyclovir 15 mg/kg/8 h (3, 5, 6)). Eighteen percent of the patients with an unknown etiology died despite this anti-infectious regimen. The possible diagnosis are numerous: pathogens not systematically looked for like West-Nile Virus, which is recognized as an emerging disease causing CNS infections in transplant recipients (23, 32, 33), other viruses (3, 22, 26, 34), auto-immune diseases including paraneoplastic and post-infectious meningoencephalitis (35), undiagnosed fungal or parasitic infections, and undiagnosed neuro-meningeal tuberculosis. Metagenomic next-generation sequencing (mNGS) (36-38) should be used for an unbiased pathogen detection for cases of unknown origin as well as *M. tuberculosis* MTB/RIF Xpert^®^ PCR testing (39).

*C. neoformans* was by far the first cause of M/ME (41 cases, 20% of all M/ME). This is consistent with *C. neoformans* infection being previously reported as the third-most frequent invasive fungal infection in a large mixed cohort of SOTRs (40). *C. neoformans* was also described as a rising pathogen in the SOTR population (41, 42) with a dose-dependent association with T-depleting induction treatments (43). This particularly high number of cryptococcal infections in M/ME is not surprising, given the tropism of *C. neoformans* for the CNS (where there is no cellular immunity) of immunocompromised hosts and the putative association of cryptococcal infections with chronic kidney failure (9, 42-44). The recently published series of CNS infections in SOTRs showed more Aspergillus than *Cryptococcus* infections: the difference can be explained by the inclusion of brain-space occupying lesions in that study, the inclusion of other types of SOTs or to antifungal prophylaxis, apparently given to a significant number of patients in this cohort (21). *C. neoformans* infection occurred at all times after KT consistent with a primary infections from the environment where it is ubiquitous (42, 45). *C. neoformans* should be specifically and systematically tested for in any case of neurological event in KTRs ([46) given the variability of the biological characteristics such as the cellularity of the CSF that can range from acellular to profuse pleocytosis of any type. Direct examination with India ink staining has a low sensitivity in SOTRs due to a weak fungal load (47). CrAg testing should be performed both in the peripheral blood and in the CSF in case of suspicion.

CNS infections caused by filamentous fungi were associated with an early occurrence and a very poor outcome, as already reported (9, 12, 21). All the cases also presented another site of infection that could help with the diagnosis as previously reported (21).

VZV infection always presented as lymphocytic meningo-encephalitis, with an external (skin or eye) simultaneous recurrence in two-thirds of the cases. In VZV-seronegative KT candidates, VZV live-attenuated vaccine represents a very effective prevention and should be considered according to the guidelines (48, 49).

Bacterial meningitis was mostly due to *Mycobacterium tuberculosis*, GNRs and Staphylococci.

Interestingly, and for the first time, GNRs, Enterobacterales, were found as the first cause of pyogenic meningitis. GNR meningitis mostly occurred in the first 5 years after KT and manifested as a critical disease with a very high 1-year mortality. Half of the cases were not secondary to a urinary, blood or digestive infection. There was no case suspect of strongyloidiasis (all patients treated before transplantation with Ivermectin, no eosinophilia, no pulmonary involvement). We hypothesize that gut microbiota alterations in the pre- and post-transplantation setting can play a role. The combination of antibiotic treatment and the direct effects of immunosuppressive drugs on the gut microbiota result in an increase in proteobacteria (50, 51) associated with the development of infections, by immune dysregulation and the promotion of virulent strains (8, 52).

The CSF-to-blood glucose ratio did not appear as a reliable tool to identify bacterial M/ME. This may be explained by the frequency of fungal M/ME in immunosuppressed hosts as opposed to the general population. However, this ratio was frequently lower than 0.5 in patients suffering from a bacterial or a fungal M/ME.

Because third-generation cephalosporins and amoxicillin, recommended as a probabilistic therapy in case of M/ME in an immunocompromised population (5,6) do not cover ESBL-producing GNRs, *Staphylococci* nor *Enterococcus faecium*, we suggest a probabilistic therapy with drugs with a good blood barrier penetration, consisting in an association of high-dose parenteral meropenem and linezolid, that would cover all the rapid-growth bacteria not sensitive to the combination recommended in the guidelines. These antibiotics also cover *S. pneumoniae*, *N. meningitidis*, *L. monocytogenes*, and *H. influenzae* M/ME.

The interest of dexamethasone in KTRs MME is limited to *S. pneumoniae* and tuberculous meningitis, and should only be used in case of a strong suspicion (i.e., compatible direct examination or recent history of a local infection) as it can be associated with a poorer outcomes in other causes (5, 7).

Survival was very heterogenous depending on the diagnosis. Some bacterial (*Staphylococci* and GNRs) and some fungal (filamentous fungi) M/MEs were associated with the highest mortality and should be considered as the principal threat in case of M/ME in KTRs.

Some limitations should be however taken in consideration for external validity: first this study only included patients from France and some etiologies of M/ME tend to vary from one country to another (for instance tick-borne, Japanese, or Saint-Louis encephalitis are not to barely present on mainland France), second, the immunosuppression strategies can differ from one country to another and may result in a different epidemiology, and third the absence of a control group prevented us from identifying risk factors.

## Conclusion

M/ME after KT encompass a wide range of causative diagnoses, mechanisms, and outcomes, of which our study provides a very detailed view. We show that the recommendations for the management of M/ME in the general population cannot be applied to KTRs in France. We believe that further studies should be performed in order to build specific guidelines for the management of M/ME in SOTRs.

## Data Availability

The original contributions presented in the study are included in the article/Supplementary Material, further inquiries can be directed to the corresponding author.
