# Supplementary material for: Specificities of Meningitis and Meningo-Encephalitis After Kidney Transplantation: A French Retrospective Cohort Study
Source: Transpl Int. 2023 Jan 18;36:10765. doi: 10.3389/ti.2023.10765 (PMC9889366; doi:10.3389/ti.2023.10765)
Supplement: Supplementary file 1 [file DataSheet1.docx]

**Supplementary data**

**Supplementary Table 1: ICD 10 codes related to meningitis and meningo-encephalitis**

| Code | Description |
| --- | --- |
| A170 | Tuberculous meningitis (G01*) |
| A321 | Listerial eningitis and meningo-encephalitis |
| A390 | Meningococal meningitis (G01*) |
| A87 | Viral meningitis |
| A870 | Enterovirus meningitis (G02.0*) |
| A871 | Adenovirus meningitis (G02.0*) |
| A872 | Lymphocytic choriomeningitis |
| A878 | Other viral meningitis |
| A879 | Viral meningitis |
| B003 | Herpesviral meningitis (G02.0*) |
| B010 | Varicella meningitis (G02.0*) |
| B021 | Zoster meningitis (G02.0*) |
| B051 | Measles complicated by meningitis (G02.0*) |
| B261 | Mumps meningitis (G02.0*) |
| B375 | Candida Meningitis (G02.1*) |
| B384 | Coccidioïdomycosis meningitis (G02.1*) |
| B582 | Toxoplasma Meningo-encephalitis (G05.2*) |
| G000 | Haemophilus meningitis |
| G001 | Pneumoccocal meningitis |
| G002 | Streptococcal meningitis |
| G003 | Straphyloccal meningitis |
| G008 | Other bacterial meningitis |
| G009 | Bacterial meningitis |
| G01 | Meningitis in bacterial diseases classified elsewhere |
| G020 | Meningitis in other infectious and parasitic diseases classified elsewhere |
| G021 | Meningitis in other mycosis classified elsewhere |
| G028 | Meningitis in other infectious and parasitic diseases classified elsewhere |
| G030 | Nonpyogenic meningitis |
| G031 | Chronic meningitis |
| G032 | Benign recurrent meningitis [Mollaret] |
| G038 | Meningitis due to other specified causes |
| G039 | Meningitis, unspecified |
| G040 | Acute disseminated encephalitis and encephalomyelitis (ADEM) |
| G041 | Tropical spastic paraplegia |
| G042 | Bacterial meningoencephalitis and meningomyelitis, not elsewhere classified |
| G048 | Other encephalitis, myelitis and encephalomyelitis |
| G049 | Encephalitis, myelitis and encephalomyelitis, unspecified |
| B451 | Cerebral cryptococcosis |

ICD-10 codes related to kidney transplantation

| Codes | Description |
| --- | --- |
| Z940 | Kidney transplant status |
| T86.1 | Complications of kidney transplant |

**Supplementary Table 2: Number of cases per etiology**

| **Cause** | Nb cases (%), N=199 |
| --- | --- |
| *Cryptococcus neoformans* | 41 (20.5) |
| Unknown | 29 (14.5) |
| Varicella-Zoster virus | 27 (13.5) |
| *Mycobacterium tuberculosis* | 11 (5.5) |
| *Enterobacteriales* | 9 (4.5) |
| Post-transplant lymphoproliferative disorder (including EBV-related forms) | 8 (4.0) |
| *Toxoplasma gondii* | 7 (3.5) |
| *Aspergillus spp.* | 6 (3.0) |
| Enterovirus | 6 (3.0) |
| *Staphylococcus aureus* | 5 (2.5) |
| *Herpes-simplex virus* | 5 (2.5) |
| *Listeria monocytogenes* | 5 (2.5) |
| *Intravenous immunoglobulins* | 5 (2.5) |
| *Streptococcus pneumoniae* | 5 (2.5) |
| Cytomegalovirus | 4 (2.0) |
| Epstein-barr virus | 4 (2.0) |
| *Borrielia budgorferi* | 3 (1.5) |
| *Nocardia* | 3 (1.5) |
| Coagulase-negative *Staphylococci* | 2 (1.0) |
| Enterococcus | 2 (1.0) |
| *Hemophilus influenzae* | 2 (1.0) |
| Neoplastic (melanoma, pulmonary adenocarcinoma) | 2 (1.0) |
| *Rhizopus spp.* | 2 (1.0) |
| Hepatitis E Virus | 1 (0.5) |
| Human immunodeficiency virus | 1 (0.5) |
| Rift Valley fever | 1 (0.5) |
| West Nile virus | 1 (0.5) |
| Tacrolimus | 1 (0.5) |
| Rapamycin | 1 (0.5) |
| Unknown bacterium | 1 (0.5) |

**Supplementary Table 3: Extra-neurologic involvement in the main causes of M/ME.**

| Cause | Nb of occurrence/Nb of cases (%) | Involved organs |
| --- | --- | --- |
| *Cryptococcus neoformans* | 19/41 (46) | Blood (9), Lung (6), Skin (3), Kidney 2), Heart (1) |
| Varicella-Zoster Virus | 18/27 (66) | Skin (13), Peripheral nervous system (4), Eye (2) |
| *Mycobacterium tuberculosis* | 5/11 (45) | Lung (4), Heart (2), Peripheral nervous system (1) |
| Gram-negative rods | 5/9 (55) | Urinary tract (3), Blood (2) |
| *Aspergillus spp.* | 6/6 (100) | Lung (3), Sinus (2), Bone (1) |
| *Rhizopus spp.* | 2/2 (100) | Sinus (1), Lung (1) |
| *Staphylococcus spp.* | 6/7(86) | Blood (7), Heart (3) |
| Toxoplasmosis | 2/7 (29) | Muscle (2), Lung (1) |
| HSV (3/5) | 3/5 (60) | Skin (3) |
| CMV | 3/4 (75) | Bowel (2), bone marrow (1) |

**Supplementary Table 4: Distribution of the ten main causes (N≥6) of M/ME according to the immunosuppression status**

| **Cause** | Highly immunocompromised  (N=91) | Non Highly  immunocompromised  (N=103) | *p* |
| --- | --- | --- | --- |
| *Cryptococcus neoformans* | 17 (18.6) | 24 (23.3) | 0.04 |
| Unknown | 15 (16.1) | 14 (13.6) |  |
| Varicella-Zoster virus | 13 (14.3) | 14 (13.6) |  |
| *Mycobacterium tuberculosis* | 3 (3.2) | 8 (7.8) |  |
| Post-transplant lymphoproliferative disorder (including EBV-related forms) | 3 (3.2) | 5 (4.9) |  |
| *Toxoplasma gondii* | 4 (4.3) | 3 (2.9) |  |
| *Enterobacteriales* | 5 (5.4) | 4 (3.8) |  |
| *Filamentous fungi* | 8 (8.8) | 0 (0) |  |
| *Staphylococcus* | 3 (3.2) | 7 (6.8) |  |
| Enterovirus | 4 (4.3) | 2 (1.9) |  |
